# 1796. Evaluation of Asymptomatic Bacteriuria in Critical Access Hospitals

**DOI:** 10.1093/ofid/ofac492.1426

**Published:** 2022-12-15

**Authors:** Whitney Hartlage, Jeannie D Chan, Natalia Martinez-Paz, John B Lynch, Rupali Jain, Paul Pottinger, Chloe Bryson-Cahn, Alyssa Y Castillo, Alyssa Y Castillo, Zahra Kassamali-Escobar

**Affiliations:** Harborview Medical Center/UW Medicine, Seattle, Washington; Harborview Medical Center, Seattle, Washington; University of Washington Tele-Antimicrobial Stewardship Program, Seattle, Washington; Harborview Medical Center, Seattle, Washington; University of Washington Medical Center-Montlake, Seattle, WA; University of Washington Medical Center-Montlake, Seattle, WA; Harborview Medical Center, Seattle, Washington; UW Medicine, Seattle, Washington; UW Medicine, Seattle, Washington; UW Medicine Valley Medical Center, Seattle, Washington

## Abstract

**Background:**

The University of Washington Tele-Antimicrobial Stewardship Program (UW-TASP) provides antimicrobial stewardship education and training to rural and critical access hospitals (CAHs) in the United States through collaborative tele-mentoring. In 2021, UW-TASP implemented a pilot stewardship cohort to reduce antibiotic treatment of asymptomatic bacteriuria (ASB). We sought to quantify the overall prevalence of ASB and proportion treated in participating hospitals.

**Methods:**

Patients undergoing urine testing were identified through local electronic medical records and microbiology data. CAHs adjudicated their own cases and reported demographics, symptoms of urinary tract infection, systemic inflammatory response symptoms (SIRS), location at the time of culture, laboratory results, and antibiotic treatment through a RedCap collection tool. The data form was created and analyzed by UW-TASP faculty. This study was waived by the University of Washington institutional review board.

**Results:**

Nineteen CAHs in 5 states participated in this pilot. Eight submitted urine analysis and culture data for 417 patients. Seventy-seven percent of patients were female and the median age was 70. The emergency department was the most common culture collection location (274/417, 66%), followed by ambulatory care clinics (111/417, 27%), and nursing facilities (13/417, 3%). SIRS criteria and/or organ dysfunction were present in 149/417 patients (36%). Two hundred sixty patients (62%) had a positive culture with Escherichia coli being the most common implicated organism (167/260, 64%). ASB was identified in 69/260 patients (27%), and antibiotics were prescribed for 53/69 (77%) of those with ASB. Oral antibiotics were prescribed for 311 (75%) patients. Of those, 22% were prescribed a fluoroquinolone. Median treatment duration was 7 days (range, 1-14).

**Conclusion:**

Although the prevalence of ASB was only 27% among 417 patients, treatment of ASB was high at 77%. High numbers of culture collections from the emergency department and ambulatory care settings identify these locations as future foci for stewardship interventions in CAHs. Low hanging fruit for intervention include reducing unnecessary fluoroquinolone use and reducing duration of antibiotic therapy.

**Disclosures:**

**Chloe Bryson-Cahn, MD**, Alaska Airlines: Advisor/Consultant.

